# THBS1 Is a Novel Serum Prognostic Factors of Acute Myeloid Leukemia

**DOI:** 10.3389/fonc.2019.01567

**Published:** 2020-02-07

**Authors:** Lidan Zhu, Qiong Li, Xiaoguo Wang, Jun Liao, Wei Zhang, Lei Gao, Yao Liu, Cheng Zhang, Xi Zhang, Jun Rao, Peiyan Kong

**Affiliations:** ^1^Medical Center of Hematology, Xinqiao Hospital, Army Medical University, Chongqing, China; ^2^State Key Laboratory of Trauma, Burns and Combined Injury, Army Medical University, Chongqing, China

**Keywords:** Acute myeloid leukemia, protein microarray, serum protein markers, THBS1, promoter methylation

## Abstract

Dysregulation of cytokines and growth factors is a general feature of tumor microenvironment, and unraveling the expression spectrum of cytokine and growth factor in niche is of utmost importance. Here, we evaluated cytokine profiling of bone marrow serum samples in AML patients and healthy controls. Protein expression profiling of serum from nine AML patients and five healthy controls was obtained using a biotinylated antibody chip. A total of 507 cytokines and growth factors were analyzed. Compared with healthy people, AML patients expressed 31 signature proteins, among which, 27 were significantly higher expressed and 4 proteins were lower. When patients were divided into favorable and poor prognosis, 12 signature proteins were significantly differentially expressed between these two groups. Furthermore, in order to identify the accuracy of cytokine expression profiles, we verified and analyzed the expression of THBS1 (Thrombospondin 1) in 116 patients and 9 healthy people. We found that THBS1 was lowly expressed in AML patients, which might be induced by promoter methylation, and patients with low THBS1 possessed shorter survivor time. Our data indicated that we successfully unveil differentially expressed proteins in AML patients using a biotinylated antibody chip; among them, THBS1 may be a potential therapeutic target for AML patients' treatment.

## Introduction

Acute myeloid leukemia (AML) is a phenotypic and genetically heterogeneous hematological disease, curable in 30–50% of younger patients (1, 2). Cytogenetic and molecular genetic detection has become the main basis in the clinical risk classification of AML (3, 4). In recent years, many mutations have been identified in leukemia cells, such as recurrent lesions in the nucleophosmin gene (*NPM1*), *FLT3-ITD, CEBPA, TET2*, etc. It has been demonstrated that the extensive genetic abnormalities play an important role in the diagnosis, risk assessment, and determination of the prognosis of patients. According to the United States National Comprehensive Cancer Network (NCCN) guideline, the risk classification of AML can be divided into favorable, intermediate, and poor prognosis (5). However, in real clinical practice, many patients with favorable prognosis showed low sensitivity or no response to standard treatment strategy. Therefore, further investigation of risk classification biomarker is needed.

Accumulating evidence indicates that tumor growth and progression are dependent on the malignant potential of tumor cells and factors secreted by the cells from the tumor microenvironment. Xenograft leukemia models demonstrated that leukemia cells often reside and engraft in normal endosteal niches, and these leukemia cells were quiescent and resistant to standard chemotherapy (6, 7). Other studies found that AML bone marrow (BM) niches are dynamically changed, leukemia cell dissemination could damage normal BM microenvironments, and the extent of the damage varied with cells engraftment; in addition, reconstruction of new niche benefit for leukemia cell growth could also be observed, and this finding demonstrated that AML BM niche played a critical role for tumor cell proliferation and expansion (8). Therefore, exploring the component of BM microenvironment and the interaction of leukemia cells and stromal cell was important for unveiling the mechanism of leukemogenesis.

Protein chip is a proteomic research tool to elucidate the expression pattern of cytokines and growth factor in tumor microenvironment (9, 10). In the present study, we show the results of a comprehensive analysis of 507 cytokines and growth factors in bone marrow serum of AML patients and healthy people using a biotinylated antibody chip. When patients were divided into a favorable and a poor prognosis group, 12 signature proteins were significantly and differently expressed. Compared with healthy people, 31 signature proteins were found to be expressed differently in AML patients; among these proteins, four were lowly expressed, including THBS1, CXCL4, MMP3, and SPARC. THBS1 is an adhesive glycoprotein that mediated cell-to-cell and cell-to-stromal interaction and is associated with platelet aggregation, angiogenesis, and tumorigenesis (11–13). Furthermore, in order to identify the results of expression profiles, we verified and analyzed the expression of THBS1 in the 116 patients and 9 healthy controls. We found that THBS1 was lowly expressed in AML patients, which might be induced by promoter methylation, and patients with low THBS1 expression possessed shorter survivor time; furthermore, allogenic hematopoietic stem cell transplantation could conquer the bad effect mediated by THBS1 low expression.

## Materials and Methods

### Human Bone Marrow Samples and Cell Culture

The bone marrow serum samples obtained from nine newly diagnosed AML and five healthy controls were used for RayBiotech biotinylated antibody chip analysis. Information about AML diagnosis, staging, histology, grade, and age was available to the investigators. All serum samples were aliquot and stored at −80°C until use. This study was approved by the Xinqiao Hospital Ethics Committee and all patients provided a signed informed consent.

From August 2011 to October 2012, 116 AML (non-APL) newly diagnosed patients admitted at Xinqiao Hospital were used to evaluate the expression of THBS1. All experiments involving human specimens were approved by the Ethics Committee of Army Medical University. The patients were followed up at least 5 years after diagnosis.

The human AML cell lines HL-60, SKM-1, and MV4-11 were obtained from the American Type Culture Collection (ATCC) (Vienna, VA). HL-60/ADM were purchased from the Institute of Hematology, Chinese Academy of Medical Sciences. Cells were cultured in Iscove's Modified Dulbecco's Medium (IMDM) (Gibco, CA) containing 10% fetal bovine serum (FBS) (Gibco, CA) and were incubated at 37°C in a humidified incubator with 5% CO_2_.

### ELISA Assay

The serum level of THBS1 in AML patients and healthy controls was examined by RayBio Human Thrombospondin-1 ELISA Kit (RayBiotech, Norcross, GA). The detailed assay was conducted according to the kit's standard procedure, and each experiment was repeated independently three times.

### Methylation Analysis

DNA was extracted using a Wizard Genomic DNA purification kit (Promega A1120). The methylated primers of methylation-specific PCR were designed using Methyl Primer Express version 1.0 software for detecting the methylated CpG islands upstream form the THBS1; the methylated-specific primers for THBS1 are as follows: forward primer: 5′-TGCGAGCGTTTTTTTAAATGC-3′, reverse primer: 5′-TAAACTCGCAAACCAACTCG-3′. Unmethylated primers for THBS1 are as follows: forward primer: 5′-GTTTGGTTGTTGTTTATTGGTTG-3′, reverse primer: 5′-CCTAAACTCACAAACCAACTCA-3′. In brief, after DNA of AML cell lines and AML patients' specimens were extracted, according to the procedure of the EZ DNA Methylation^TM^ Kit (Irvine, CA), bisulfite modified DNA was isolated; after amplification, PCR products were separated on agarose gel and visualized by ethidium bromide fluorescence using the LAS-1000 Imager.

### Data Resource and Preprocessing

The expression profiling of MILE study (Microarray innovations in leukemia) was downloaded from the GEO dataset (GSE13164); 58 healthy control and 257 AML samples were included. Two gene methylation profiling datasets were also downloaded from the GEO dataset (GSE40871, GSE80762); GSE40871 was designed for evaluation genomic impact of transient low-dose decitabine treatment on primary AML cells, and GSE40871 was designed for global DNA methylation of AML treated with decitabine 20 mg/m^2^/day on days 1–10. The two datasets were tested on the platform of GPL13534.

### Antibody Chip Technology

Before the experiments, the overall sensitivity of the RayBio^®^ Human Label-based Arrays was evaluated, the detection dynamics ranged from 5 to 1,000 pg/ml. The detection sensitivity for individual proteins varied and depended mainly on the binding affinity. Nevertheless, a linear increase in spot intensity was observed with the concentration of all the proteins that was tested. In order to test the specificity of the arrays, one dozen recombinant proteins were individually labeled. Then, labeled proteins were incubated with the arrays at a final concentration of 100 ng/ml. Individual biotin-labeled protein mainly bound to the spot where its corresponding antibody was printed, even at high concentrations, suggesting the high specificity of the arrays. No signal was detected when a biotin-labeled solvent was used. These results demonstrate the specificity of our system.

### Statistical Methods

All data were analyzed using SPSS 17.0 software. The correlation of THBS1 with clinicopathologic features of patients was accessed by the Pearson χ^2^-test. Survival estimates were obtained by using Kaplan–Meier method, and comparisons were made by log-rank test; when analyzing the survival difference of THBS1^High^ patients and THBS1^Low^ patients, patients submitted to bone marrow transplant were not included. Unpaired Student's *t*-test for two groups and one-way ANOVA for multiple group data were applied in this study. A COX proportional regression model was used to calculate the survival hazard ratio (HR). Statistical difference was considered significant if *P* < 0.05.

## Results

### Patient Characteristics

For patients who underwent protein chip analysis, patients' demographics, and clinical characteristics are summarized in Table S1. Among these specimens, the age of nine newly diagnosed AML patients ranged between 18 and 56 years, with a mean age of 38.7 years; the age of healthy people ranged from 24 to 47 years, with a mean age of 37.2 years old. According to the NCCN guideline, the favorable and poor prognosis groups were stratified. Four patients were identified as part of the favorable prognosis group, and five patients were classified into the poor prognosis group. In patients who underwent verification of THBS1 expression, patients' demographics, and clinical characteristics are summarized in Table 1, which shows that THBS1 expression was significantly correlated with patients' cytogenetics abnormality and bone marrow blasts, but not with the gender, age, and FAB classification, as well as median WBC count, Hb concentration, and platelet count.

**Table 1:** Correlation between THBS1 protein and clinicopathological features of AML patients.

**Prognostic variables**	**No**.	**Serum THBS1 expression**			
		**Low**	**High**	**χ*^**2**^***	***P*-value**
**GENDER**					
Female	52	24	28	0.183	0.669
Male	64	27	37		
**AGE**					
>38	61	29	32	0.668	0.414
≤38	55	22	33		
**FAB CLASSIFICATION**					
M0	9	4	5	3.017	0.807
M1	19	10	9		
M2	32	11	21		
M3	0	0	0		
M4	26	13	13		
M5	18	9	9		
M6	12	4	8		
**CYTOGENETICS ABNORMALITY**					
Favorable	12	2	10	7.922	0.019
Intermediate	40	14	26		
Poor	64	35	29		
Median WBC (× 10^9^, range)	46.8 (0.53–365.29)		45.7 (0.6–380)		0.521
Median Hb concentration (g/dL, range)	74.1 (31-126)		76.5 (32-134)		0.711
Median platelet count (× 10^9^, range)	57.4 (2-280)		62.4 (3-222)		0.402
Bone marrow blast (%, range)	69 (21-99)		59 (20-96)		0.015

### Analysis of Serum Cytokine Profiling of AML Patients and the Healthy People

**Figure 1:**
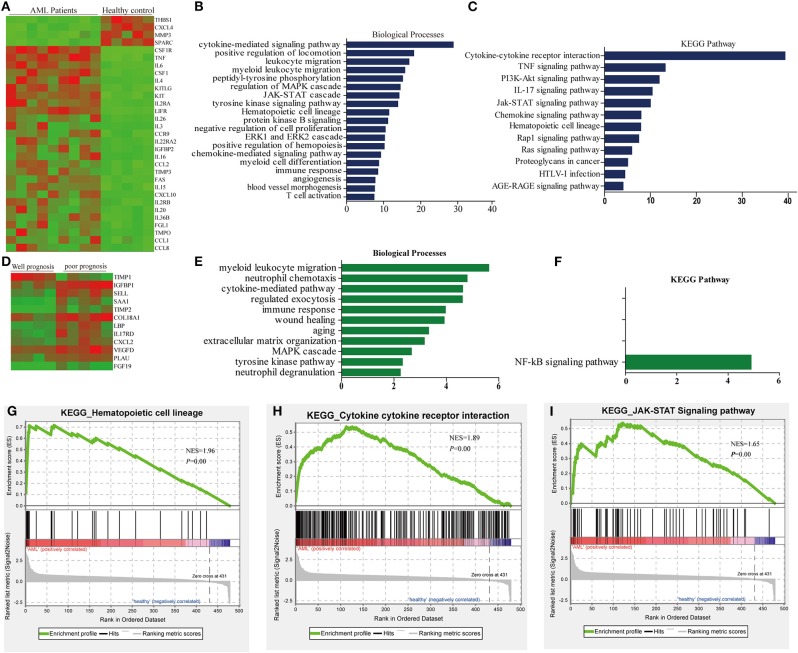
Differential abundance of serum cytokines in AML patients and normal controls. **(A)** Hierarchical clustering analysis of cytokine profiles based on the top 31 genes in AML patients and healthy controls. **(B)** Biological process revealed the significant association of the genes with different expression in each group, column length: –log(P). **(C)** Heatmap of Kyoto Encyclopedia of Gene and Genomes (KEGG) enriched terms colored by *P*-values. **(D)** Hierarchical clustering analysis of cytokine profiles based on the top 12 genes in AML patients. **(E)** Biological process enriched terms colored by *P*-value, column length: –log(P). **(F)** KEGG enriched terms colored by *P*-values. **(G–I)** The top three enriched pathway in AML patients and healthy control analyzed by gene set enrichment analysis (GSEA).

A quantitative comparison of the 507 cytokines and growth factors between AML patients and healthy people bone marrow serum was performed. The amount of each sample was normalized based on total protein concentration. Among the 507 cytokines, 64 cytokines, and growth factor were found expressed significantly and differentially, and 31 cytokines were changed more than 2-fold (Table S2). Twenty-seven were found significantly higher expressed, such as CSF1R, TNF, IL6, CSF1, IL4, KITLG, KIT, IL2RA, LIFR, IL26, IL3, CCR9, IL22RA2, IGFBP2, IL16, CCL2, TIMP3, FAS, IL15, CXCL10, IL2RB, IL20, IL36B, FGL1, TMPO, CCL1, and CCL8. Four cytokines of AML patients' serum were lower: CXCL4, MMP3, THBS1, and SPARC; the expression patterns of cytokines were summarized by using hierarchical clustering analysis (Figure 1A). Then, we performed the gene enrichment analysis using the Metascape database (http://metascape.org); we found that the biological processes were significantly enriched in the AML patients, such as cytokine-mediated signaling, positive regulation of locomotion, leukocyte migration, peptidyl-tyrosine phosphorylation, regulation of MAPK cascade, JAK-STAT cascade, tyrosine kinase signaling pathway, etc. (Figure 1B). In addition, the KEGG pathway was significantly enriched in the AML group (Figure 1C), such as cytokine–cytokine receptor interaction, TNF signaling pathway, PI3K-Akt signaling pathway and IL17 signaling, JAK-STAT signaling pathway, Rap1 signaling pathway, etc.

According to the clinicopathological parameters and cytogenetic abnormalities of the newly diagnosed AML patients, the patients were divided into favorable prognosis and poor prognosis group. Twelve cytokines were found to be expressed significantly and differentially. Among these cytokines, 11 cytokines of the poor prognosis group were higher than that of the favorable prognosis group, such as TIMP2, FGF19, IL17RD, SAA1, COL18A1, PLAU, CXCL2, LBP, SELL, IGFBP1, and VEGFD (Figure 1D, Table S3). GO and KEGG analysis in the Metascape database were also predicted, and biological processes, such as myeloid leukocyte migration, neutrophil chemotaxis, etc., were enriched in the poor prognosis group (Figure 1E), but only the NF-KB signaling pathway could be enriched significantly in KEGG analysis (Figure 1F).

Next, gene enrichment analysis was further validated by Gene set enrichment analysis (GSEA), and the results revealed that only hematopoietic cell lineage, cytokine–cytokine receptor interaction, and JAK-STAT signaling pathway could be enriched significantly (Figures 1G–I); this might be due to the small sample size included. However, the results of GSEA analysis highly overlapped with the results of GO analysis; cytokine–cytokine receptor interaction and JAK-STAT signaling pathway could be enriched significantly in both methods, suggesting that these two pathways might play an important role in tumorigenesis of AML.

### High Serum Expression THBS1 Is Associated With Longer Survival of the Patients

**Figure 2:**
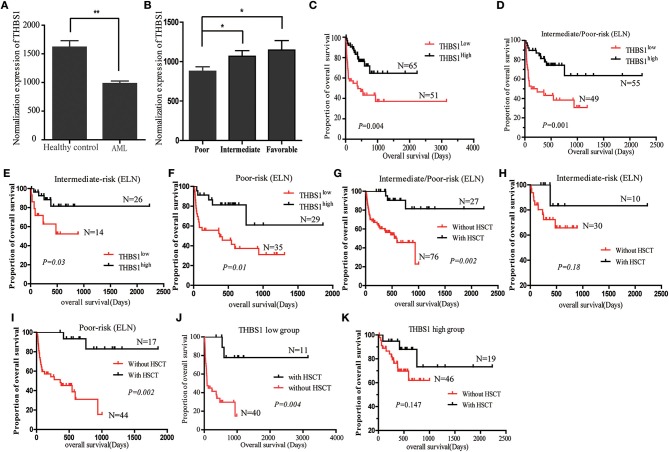
Expression of THBS1 in the AML patients and normal control serum samples. **(A)** Expression level of THBS1 in 116 AML patients and 9 healthy controls. **(B)** Serum expression of THBS1 in AML patients with favorable, intermediate, and poor prognosis. **(C)** Kaplan–Meier survival curves estimated overall survival of patients with different expression of THBS1. **(D–F)** Kaplan–Meier estimated overall survival of serum THBS1 expression in patients with intermediate/poor prognosis, intermediate prognosis, and poor prognosis group. **(G–I)** Kaplan–Meier survival curves estimated overall survival of treatment with HSCT in patients with intermediate/poor prognosis, intermediate prognosis, and poor prognosis group. **(J,K)** Overall survival analysis of HSCT treatment in patients with high and low serum THBS1 expression patients. *P < 0.05, **P < 0.01.

The interaction of these 31 differently expressed factors was analyzed by using the STRING 9.1 database (http://string-db.org/); the protein interaction network diagram showed that THBS1 is located at the core of the interaction network diagram, suggesting that THBS1 might play a critical role in the tumorigenesis of AML. To further identify the accuracy of the protein chip results, ELISA was performed in the AML patients and healthy people's bone marrow samples. The THBS1 protein concentration in 116 AML patients was 982.78 ± 188.39 ng/ml and 1610.14 ± 206.33 ng/ml in the healthy people; the difference was statistically significant (Figure 2A). In the AML patients, no significant differences were found with respect to the age at diagnosis, gender, FAB classification, WBC at diagnosis, Hb concentration, and median platelet count in the THBS1 high and low expression group. THBS1 expression was significantly correlated with cytogenetics abnormality and bone marrow blast at diagnosis (Table 1). When these patients were divided into favorable, intermediate prognosis, and poor prognosis group, serum THBS1 level of the poor prognosis group was significantly lower than that of the favorable and intermediate prognosis groups (Figure 2B). Kaplan–Meier survival curves showed that the overall survival of patients with high THBS1 expression was significantly superior to those with low THBS1 expression (Figure 2C, *P* = 0.004). Moreover, the intermediate/poor, intermediate, and poor prognosis sub-populations, patients with higher THBS1 expression also showed longer survival times (Figures 2D–F), suggesting that THBS1 might be a prognostic biomarker for AML patients. Allogeneic hematopoietic stem cell transplantation (allo-HSCT) can significantly reduce leukemia residual disease and has been conferred a cure on many AML patients (14, 15). To investigate whether allo-HSCT had survival benefit in our cohort, Kaplan–Meier survival analysis was performed; in patients from the intermediate/poor and poor prognosis group, patients who underwent allo-HSCT had a significantly better OS, but in the intermediate prognosis subgroup, due to the sample size, no significant difference was obtained (Figures 2G–I). For patients with low THBS1 expression, we found that patients who underwent allo-HSCT showed longer survival time than patients with chemotherapy alone, and the difference was significant (Figure 2J, *P* = 0.004), but this difference could not obtained in the patients from the THBS1 high expression group (Figure 2K, *P* = 0.147), suggesting that allo-HSCT might be an optimal therapy strategy for patients with low THBS1 expression.

The results of univariate and multivariate analysis for risk factor of OS in patients are summarized in Table 2. Multivariate analysis revealed that treatment with allo-HSCT and serum THBS1 expression were independent prognostic indicators of the overall survival of patients.

**Table 2:** Univariate and multivariate analyses of the overall survival of patients.

**Prognostic variables**	**Univariate analysis**		**Multivariate analysis**	
	**HR (95% CI)**	***P*-value**	**HR (95% CI)**	***P*-value**
Gender (female vs. male)	1.01 (0.551–1.852)	0.975	0.861 (0.455–1.629)	0.645
Age (≥38 vs. <38)	1.875 (1.004–3.501)	0.049	1.761 (0.915–3.387)	0.090
WBC (≥30 vs. <30 × 10^9^/L)	1.871 (1.019–3.435)	0.043	1.627 (0.781–3.389)	0.138
Hb concentration (≥75 vs. <75g/dL)	1.026 (0.560–1.880)	0.934	0.954 (0.494–1.841)	0.843
Platelet count (≥60 vs. <60 × 10^12^/L)	0.979 (0.501–1.914)	0.951	1.558 (0.781–3.106)	0.434
Treatment with allo-HSCT (with vs. without)	0.092 (0.022–0.386)	0.001	0.088 (0.020–0.383)	0.02
THBS1 (High vs. Low)	0.336 (0.178–0.633)	0.001	0.388 (0.169–0.627)	0.001

### Promoter Methylation Contributed to Low Serum THBS1 Expression of AML Patients

**Figure 3:**
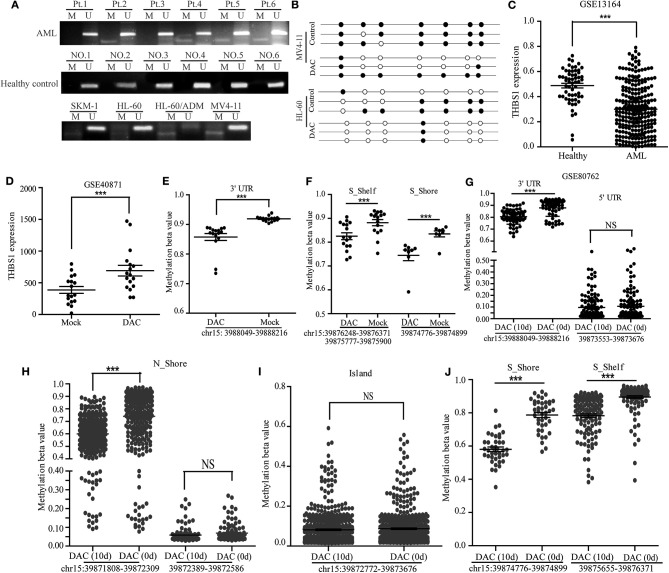
Methylation of THBS1 in AML patients and normal controls. **(A)** MSP analysis of THBS1 methylation in AML patients and healthy controls, and AML cell lines. M and U represent MSP results using primer for methylated and unmethylated THBS1 genes, respectively. **(B)** The methylation status of CpG island in the THBS1 gene promoter of MV4-11 and HL-60 cells; each row of circles represents a single clone, and open circles represent unmethylated cytosine and filled circles represent methylated cytosine. **(C)** Expression of THBS1 in AML patients and healthy controls based on the GSE 13164 dataset. **(D)** Expression of THBS1 in AML primary cells treated with decitabine (DAC) based on dataset GSE40871. **(E)** Methylation beta value of THBS1 3′-UTR in AML primary cells treated with decitabine (DAC) based on dataset GSE40871. **(F)** Methylation beta value of THBS1 S-Shelf and S-Shore in AML primary cells treated with decitabine (DAC) based on dataset GSE40871. **(G)** Methylation beta value of THBS1 3′-UTR, 5′-UTR in AML patients treated with decitabine (DAC) based on dataset GSE80762. **(H)** Methylation beta value of THBS1 N-Shore in AML patients treated with decitabine (DAC) based on dataset GSE80762. **(I)** Methylation beta value of THBS1 Island in AML patients treated with decitabine (DAC) based on dataset GSE80762. **(J)** Methylation beta value of THBS1 S-Shore, S-Shelf in AML patients treated with decitabine (DAC) based on dataset GSE80762. ns, no significant; **P < 0.01.

The abnormal methylation of the gene promoter region could lead to tumor suppressor gene inactivation and the partial activation of oncogenes. Some studies had shown that the CpG Island of the THBS1 promoter region had abnormal methylation in oral cancer, prostate cancer, lung cancer, and other tumors. We further explored the methylation level of THBS1 in our cohort, and we found that a proportion of AML patients (11/30) presented methylation, and serum samples of healthy controls did not exhibit THBS1 promoter methylation (Figure 3A). Next, we analyzed the methylation of the THBS1 promoter in AML cell lines; the methylation of THBS1 in these cell lines could be observed (Figure 3A, lower lane). In addition, bisulfite sequencing PCR was used to evaluate the methylation status of HL-60 and MV4-11 cells with DAC treatment, and the results demonstrated that fewer methylated CpG sites were found in cells with DAC treatment (Figure 3B), suggesting that demethylation of THBS1 gene could be induced by DAC treatment. In order to verify our results, we compared the expression levels of THBS1 in AML patients and healthy control samples using the GSE13164 database; THBS1 was significantly lowly expressed in the PBMC of AML patients (Figure 3C), suggesting that low serum THBS1 expression might be due to the reduced secretion of PBMC. The methylation levels were evaluated through two GEO databases (GSE40871 and GSE80762). In the GSE40871 database, when primary AML cells were treated with short-term decitabine (DAC, 100 nM, 3 days), RNA-sequencing results showed that THBS1 expression could be up-regulated significantly (Figure 3D); further genome methylation profiling array demonstrated that after cells were treated with DAC for 3 days, the methylation beta value of THBS1 3′-UTR (chr15: 3988049-39888216) was decreased significantly (Figure 3E), and the methylation beta value of S-Shelf and S-Shore was also significantly decreased with DAC treatment (Figure 3F). In GSE80762, patients with AML or MDS were treated with decitabine 20 mg/m^2^/day on days 1–10. Bone marrow samples were collected on day 0 and day 10, and the methylation beta value of THBS1 3′-UTR decreased significantly, but no difference was found in the 5′-UTR region (Figure 3G). In the N-Shore island, the methylation beta value of chr15:39871808-39872309 was found to be down-regulated with DAC treatment, and no change was observed in the site of chr15:39872389-39872586 (Figure 3H). Similarly, there was no difference in the island (chr15:39872772-39873676) (Figure 3I); consistent with GSE40871 database results, the methylation beta value of S-Shelf and S-shore decreased significantly with DAC treatment (Figure 3J). Taken together, all these results suggested that methylation of THBS1 gene in leukemia cell might be attributed to the low THBS1 levels of bone marrow serum.

## Discussion

The interaction of leukemia cells and the surrounding microenvironment in the bone marrow is very important for leukemogenesis; consequently, a better understanding of the cytokine expression spectrum in the BM niche could contribute to prognosis improvement and potential target investigation. The present study comprehensively carried out cytokine profiling of bone marrow serum in AML patients and healthy people. Compared with healthy controls, we found 31 significantly different expressed cytokines and growth factors. When the patients were divided into subgroups according to cytogenetics normality, 12 different expressed cytokines and growth factors were found. In order to verify the accuracy of chip results, the BM serum of 116 AML patients was used to evaluate THBS1 expression level, and we found that THBS1 might be a prognostic biomarker for AML patients.

The main aim of the current study was to characterize the differently secreted cytokines and growth factor composition of primary leukemia and healthy controls' BM in detail. This method involved semi-quantitative detection techniques, which will provide insight into whether certain proteins reside in the BM microenvironment. Through GO and KEGG analysis, the 19-item biological processes and 12 KEGG pathways were markedly enriched, including cytokine-mediated signaling, cell migration, peptidyl-tyrosine phosphorylation, hematopoietic cell lineage, JAK-STAT signaling, tyrosine kinase signaling pathway, myeloid cell differentiation, etc. In order to validate the pathway enriched by GO analysis, GSEA analysis was also conducted, hematopoietic cell lineage, cytokine–cytokine receptor interaction, and JAK-STAT signaling were enriched. All these results suggested that the cytokine in our array could reflect different BM microenvironments in AML and healthy controls; cytokine–cytokine receptor interaction and JAK-STAT signaling might play an important role in leukemogenesis and immune response, which also provides new potential target for AML therapy.

Abnormal regulation of cytokines in niche is an important feature of AML. To thoroughly understand the role of cytokines in mediating AML cell survival, Carey et al. used a unique *ex vivo* screen; cell viability was tested in the presence of 94 different cytokines, and only a few cytokines could significantly promote cell growth, including IL-1, GM-CSF, IL-3, M-CSF, G-CSF, and TNF-a. In the presence of exogenous IL-1, 67% of primary AML cells showed expansion of leukemia progenitors and 15-fold increased cell growth via activation of p38 MAPK, indicating that blockade IL-1 signaling might provide a therapeutic tool to target most parts of the AML subtypes (16). Other studies described that IL-1 predominantly enhanced the production of numerous cytokines, such as IL-6, IL-8, MCP-1, GM-CSF, G-CSF, etc. (17–21); all these findings suggested a promising role for IL-1 in mediating myeloid growth in AML. Moreover, IL-6 signaling and TNF-a signaling also make up part of the dysregulated cytokine network in AML; IL-6 is a pleiotropic multifunctional cytokine involved in the regulation of hematopoiesis and leukemic blast formation, and elevated serum IL-6 levels in AML patients had been reported by many studies (21, 22). However, whether IL-6 could be regarded as a predictive marker or therapeutic target is still controversial; in some patients, IL-6 could promote growth of blast cells and enhance G-CSF-dependent proliferation, but in other studies, this stimulation could not observed or even reduced (23–25). Consistent with IL-1 and IL-6 signaling, TNF-a was also dysregulated in AML patients, which might be a key autocrine factor for the survival and proliferation of leukemic stem cells, and NF-KB signaling and PI3K/AKt signaling were also engaged in the TNF-a-mediated leukemic clones' proliferation and drug resistance (26–28). All these findings suggested that dysregulation of cytokines is a general hallmark of AML patients; unraveling the mysteries of cytokine and growth factor interaction in the context of AML is of utmost importance.

In the present study, cytokine–cytokine receptor interaction and JAK-STAT pathway were mostly enriched through KEGG and GSEA analysis, hyperactive JAK-STAT pathway had been proven to have a critical role in the pathogenesis of leukemia, and JAK3-mediated high activity of STAT molecules was often observed in some leukemia patients. Moreover, some abnormal elevation cytokines including IL-6 and TNF-a also could lead to activation of JAK signaling (29, 30). Preclinical evaluation of JAK-STAT inhibition (such as JAK inhibitors: ruxolitinib, lestaurtinib, pacritinib, etc.) had demonstrated that this treatment strategy could inhibit AML cell proliferation; however, in some early-stage clinical trials, patients with single-agent JAK inhibitors could not achieve a promising therapy response, and this might be due to the crosstalk of JAK-STAT signaling pathway and other oncogenic pathways in the AML (31). Thus, in future clinical trials, combination of JAK inhibitor and other therapeutic strategies should be explored.

Great efforts had been made to explore new prognostic signatures for AML patients; gene mutation status had been integrated into AML risk classification in all kinds of guidelines, but in real clinical practice, many patients with favorable prognosis showed low sensitivity or no response to standard treatment strategy. Therefore, further investigation of risk classification biomarkers is needed. Recently, Beck et al. found that four lincRNAs' composed signature was an independent prognostic factor for AML patients, and the value of this criterion was also verified in four other patient cohorts; this study suggested that lincRNAs could be used to complement the AML risk classification (32). In our study, we also found that serum THBS1 might be a promising prognostic factor for patients. THBS1 is a member of the thrombospondin (TSP) family, which is a kind of adhesion protein within the matrix where platelets, endothelial cells, macrophages, monocytes, and other cells synthesize, and various proteins interact *in vivo*. Furthermore, it is involved in cell growth, migration, the formation of new blood vessels, and other life activities (33, 34). In recent years, THBS1, as a tumor suppressor gene, had gradually attracted people's attention, which could influence the growth of tumors by inhibiting angiogenesis and activating the transforming growth factor. In nude mice experiments, transfection of THBS1 could slow the growth, metastasis, and angiogenesis of breast cancer (35, 36). In addition, THBS1 could be induced by TGFB1 stimulation in oral squamous carcinoma cell (37). In order to verify the reliability of our study, we evaluated serum THBS1 levels in the bone marrow of AML patients and healthy adults by ELISA. We found that THBS1 was lower expressed in the serum of AML patients, and the reason for low THBS1 in serum was also discussed preliminarily; through MSP and GEO datasets, we found that compared with healthy control, THBS1 methylation rate was higher, and hypomethylating agent treatment could lead to up-regulation of THBS1 expression and reduction of methylation level. This indicated that the methylation of the THBS1 gene with AML and prognosis were correlated.

Due to the limited number of samples, the clinical significance of the detected factors remains to be further verified. Next, we will select higher specificity marker factors and expand the sample size to verify the obtained factor. Our goal is to produce a protein chip that can be used to determine the prognosis of AML patients clinically, in order to provide a new method for the clinical prediction of AML prognosis.

## Data Availability Statement

The datasets generated for this study can be found in the NCBI Gene Expression Omnibus (GSE141930).

## Ethics Statement

The studies involving human participants were reviewed and approved by Medical Ethics committee of second affiliated hospital of army medical university. The patients/participants provided their written informed consent to participate in this study. Written informed consent was obtained from the individual(s) for the publication of any potentially identifiable images or data included in this article.

## Author Contributions

PK conceived and designed the study. LZ, JR, and XW developed the methodology and analyzed and interpreted the data. JR wrote the manuscript. JL, WZ, QL, LG, YL, CZ, XZ, and PK reviewed and revised the manuscript.

### Conflict of Interest

The authors declare that the research was conducted in the absence of any commercial or financial relationships that could be construed as a potential conflict of interest.
