# Supplementary material for: THBS1 Is a Novel Serum Prognostic Factors of Acute Myeloid Leukemia
Source: Front Oncol. 2020 Feb 7;9:1567. doi: 10.3389/fonc.2019.01567 (PMC7020255; doi:10.3389/fonc.2019.01567)
Supplement: Supplementary file 1 [file Data_Sheet_1.pdf]

Supplementary Material

Table S1. Patient characteristics

Variables	N	Healthy control	AML patients	
			Well prognosis	Poor prognosis
Gender				
female	7	2	2	3
male	7	3	2	2
Age				
>36	8	3	2	3
<=36	6	2	2	2

Table S2. Different expression protein in AML patients and healthy controls

Protein Name	AML (*n*=9)	Normal (*n*=5)	*Fold change*	*P*
THBS1	2714.40 ±1256.01	15736.97±3542.9	0.17	< 0.01
CXCL4	1416.77±816	7381.40±1306.33	0.19	< 0.01
MMP3	481.30±242.91	1770.79±419.61	0.27	< 0.01
SPARC	1022.08±350.39	3744.66±611.14	0.27	< 0.01
CSF1R	2361.21±368.16	310.32±34.69	7.61	0.04
TNF	6988.11±1285.12	932.17±549.47	7.50	0.01
IL6	6985.02±1517.8	963.86±492.32	7.25	0.01
CSF1	13302.75±2721.75	2042.72±537.89	6.51	0.04
IL4	3710.93±1397.06	677.03±97.14	5.48	0.03
KITLG	2102.99±386.88	389.22±123.17	5.40	< 0.01
KIT	2197.03±328.98	408.82±178.88	5.37	0.02
IL2RA	9013.22±2686.71	1683.55±307.1	5.35	< 0.01
LIFR	2779.06±429.04	556.36±84.95	5.00	< 0.01
IL26	1314.54±556.61	267.37±88.55	4.92	0.04

IL3	4119.08±2867.97	984.35±219.62	4.18	0.05
CCR9	10415.03±3363.64	3067.12±1575.8	3.40	0.03
IL22RA2	1181.56±448.07	388.26±98.74	3.04	0.03
IGFBP2	712.71±328.45	237.26±70.38	3.00	0.03
IL16	428.09±245.86	152.98±42.77	2.80	< 0.01
CCL2	5753.51±1396.78	2081.60±611.6	2.76	0.05
TIMP3	1238.03±352.81	454.43±118.56	2.72	< 0.01
FAS	3359.07±669.94	1309.28±143.21	2.57	0.04
IL15	6757.29±1119.3	2639.64±906	2.56	0.01
CXCL10	714.37±242.56	279.79±25.06	2.55	0.04
IL2RB	1763.09±588.41	702.56±297.32	2.51	0.03
IL20	2553.77±885.01	1018.15±312.06	2.51	0.04
IL36B	2857.94±793.14	1195.39±325.05	2.39	0.01
FGL1	1958.83±610.59	881.22±245.14	2.22	0.03
TMPO	1680.45±781.97	794.06±170.76	2.12	0.04
CCL1	793.71±305.34	388.95±178.92	2.04	0.02
CCL8	544.91±220.07	272.12±72.35	2.00	0.02
IL1RAPL1	2032.15±848.63	1017.77±309.11	2.00	0.01
FGF7	721.13±296.49	362.46±202.07	1.99	0.02
INHBA	1170.68±492.52	602.16±123.87	1.94	0.04

KREMEN1	1328.14±375.04	702.40±213.77	1.89	0.05
FGFBP	765.37±289.68	409.03±154.73	1.87	0.05
CRIM1	1160.03±434.9	665.91±180.54	1.74	0.03
CCL17	588.53±157.46	342.28±71.27	1.72	0.04
MFRP	1268.82±385.2	751.45±243.19	1.69	0.05
IL1RAPL2	2114.87±773.4	1254.36±245.78	1.69	0.02
ACVR2B	977.34±282.68	582.86±89.55	1.68	0.04
IGFBP1	917.55±237.94	559.45±200.01	1.64	0.04
ANGPT2	1178.79±402.7	724.04±123.74	1.63	0.03
GFRA3	1652.33±565.56	1020.65±267.9	1.62	0.05
NRG2	3198.47±1100.39	1983.76±367.96	1.61	0.04
GFRA4	1649.28±557.4	1035.79±165.24	1.59	0.03
TYRO3	1076.69±304.44	677.83±139.06	1.59	0.04
MST1	1359.94±429.96	878.14±204.86	1.55	$<$ 0.01
MMP2	4937.18±1462.68	3209.62±687.46	1.54	0.03
LTA	5692.33±1498.67	3720.46±733.13	1.53	0.05
CCL21	4246.23±801.97	2820.45±206.95	1.51	0.04
MFGE8	10851.23±1155.83	7226.89±823.34	1.50	0.04
ADIPOR2	15035.13±3882.89	10040.56±1890.65	1.50	0.03
PLAUR	1050.79±298.6	707.32±151.84	1.49	0.05

ENG	1567.92±439.05	1056.83±183.55	1.48	0.03
TMEFF1	40532.49±9878.23	27418.34±6044.31	1.48	0.02
ICAM5	6198.55±1191.02	4327.53±939.26	1.43	0.03
MSTN	3129.05±680.89	2200.51±544.48	1.42	0.03
ANG	3917.19±998.02	2755.17±190.95	1.42	0.01
TNFRSF18	2885.28±631.89	2108.83±363.84	1.37	0.01
LCN1	29911.83±4756.36	22811.21±2351.31	1.31	0.04
GPNMB	8504.70±937.22	6564.07±1058.34	1.30	0.01
INSR	14302.23±1916.6	11386.02±1125.98	1.26	0.01
TRADD	8145.56±1092.11	6762.74±757.8	1.20	0.01

Table S3. Different expression protein in AML patients with well prognosis and poor prognosis

Protein Name	Well prognosis (*n*=6)	Poor prognosis (*n*=5)	Fold change	P
TIMP1	11805.03 ±1109.84	7185.49 ±1551.25	0.61	＜0.05
IGFBP1	686.88 ±122.52	1102.1 ±115.24	1.60	＜0.05
SELL	951.99 ±242.75	1784.52 ±283.75	1.87	＜0.05
SAA1	2677.4 ±653.4	6890.68 ±2085.87	2.57	＜0.05
TIMP2	207.68 ±5.58	784.14 ±321.8	3.78	＜0.05
COL18A1	423.45 ±92.76	646.42 ±95.95	1.53	＜0.05
LBP	4367.86 ±512.09	8659.06 ±2461.9	1.98	＜0.05
IL17RD	2558.63 ±581.81	4925.83 ±1332.99	1.93	＜0.05
CXCL2	5154.42 ±1021.86	7865.88 ±1536.42	1.53	＜0.05
VEGFD	2011.11 ±68.6	2615.46 ±399.24	1.30	＜0.05
PLAU	10289.22 ±3165.27	14541.45 ±713.79	1.41	＜0.05
FGF19	514.22 ±25.13	1523.58 ±550.84	2.96	< 0.01
